# Supplementary material for: An open-source framework for large-scale, flexible evaluation of biomedical text mining systems
Source: J Biomed Discov Collab. 2008 Jan 29;3:1. doi: 10.1186/1747-5333-3-1 (PMC2276192; doi:10.1186/1747-5333-3-1)
Supplement: Additional file 1 — Evaluation platform source code. The additional file contains the software evaluation framework discussed in this paper. The most current release can be downloaded from SourceForge.net [1]. See the accompanying README file for instructions on installation and use. Also included in the distribution is a collection of UIMA wrappers for some commonly used BioNLP tools and annotated corpora. [file 1747-5333-3-1-S1.zip › bionlp_uima_repository-1.0.1/docs/type_system_description/ccpTypeSystemManual.pdf]

MANUAL

Type System Manual

BioNLP UIMA Component Repository

Center for Computational Pharmacology

September 2007

The CCP UIMA Type System

The CCP Type System extends the base *uima.tcas.Annotation* class to create annotations that are both comprehensive in regards to the meta information that they store as well as flexible in terms of the types of annotations that can be created. The central class of the CCP Type System, *CCPTextAnnotation*, ties the various other supporting classes together as described below. A figure of the class hierarchy is included at the end of this document.

Text Annotation Class Descriptions

CCPTextAnnotation [edu.uchsc.ccp.uima.annotation]
The CCPTextAnnotation extends the base *uima.tcas.annotation* class to include an annotation ID, the capability for multiple annotation spans, a link to the annotator responsible for generating the annotation, membership to annotation sets, and a link to a class mention which defines the class (or type) of this annotation.

- *AnnotationID <Integer>*: The annotation ID provides a means for identifying a particular annotation. Setting this ID is optional. The default value should be set to -1.

- *AnnotationSets <FSArray:CCPAnnotationSet>*: Annotation Sets provide an arbitrary means of categorizing and clustering annotations into groups.

- *Annotator <CCPAnnotator>*: The annotator responsible for generating this annotation.

- *DocumentSectionID <Integer>*: The document section ID is optionally used to log which section of a document this annotation is in. Values can be specified by the user. See *edu.uchsc.ccp.util.nlp.document.DocumentSectionTypes* for a few common sections.

- *NumberOfSpans <Integer>*: The number of spans comprising this annotation. The CCPTextAnnotation allows for the use of multiple spans for a single annotation.

- *Spans <FSArray:CCPSpan>*: This FSArray stores the CCPSpans which comprise this annotation. It should be noted that for an annotation with multiple spans, the default *begin* and *end* fields should be set to the beginning of the first span and the end of the final span, respectively.

- *ClassMention <CCPClassMention>*: The CCPClassMention indicates the type (or class) for this annotation.

CCPAnnotationSet [edu.uchsc.ccp.uima.annotation]
The annotation set provides a means for arbitrarily categorizing or clustering groups of annotations. Annotations can be associated with multiple annotation sets. One usage example would be the creation of an annotation set to distinguish gold standard annotations for a particular document collection. Each annotation set is associated with a unique ID, a name and a description.

- *AnnotationSetID* <*Integer*>: An Integer uniquely identifying a particular annotation set.
- *AnnotationSetName* <*String*>: The name of the annotation set.
- *AnnotationSetDescription* <*String*>: A textual description of the annotation set.

CCPAnnotator [edu.uchsc.ccp.uima.annotation]
The annotator object contains information which is used to determine who/what generated an annotation. The annotator can represent a human annotator, or it can represent a tool, e.g. a POS tagger, entity tagger, etc.

- *AnnotatorID* <*Integer*>: An Integer uniquely identifying a particular annotator.
- *FirstName* <*String*>: The first name of the annotator.
- *LastName* <*String*>: The last name of the annotator.
- *Affiliation* <*String*>: The institutional affiliation of the annotator.

CCPSpan [edu.uchsc.ccp.uima.annotation]
The span object holds span information. This class was created to allow the CCPTextAnnotation to handle multi-span annotations.

- *SpanStart* <*Integer*>: The character offset for the start of the span.
- *SpanEnd* <*Integer*>: The character offset for the end of the span.

CCPSemanticAnnotation [edu.uchsc.ccp.uima.annotation]
The CCPSemanticAnnotation is the superclass for all semantic (i.e. non-syntactic) annotations.

CCPSyntacticAnnotation [edu.uchsc.ccp.uima.annotation]
The CCPSyntacticAnnotation is the superclass for all syntactic annotations.

Mention (Annotation Type) Class Descriptions

The architecture of the mention (annotation type) structure was designed to be flexible in its ability to represent virtually any frame-based class. The design mirrors the mention structure used in Knowtator[1], an annotation tool developed within the Center for Computational Pharmacology. In short, this structure is analogous to the classic frame/slot structure introduced by Minsky. The class mention (or concept mention as it is described in the Knowtator documentation) can be thought of as a frame. It represents the semantic type of an annotation. Examples of class mentions include, but are not limited to, things such as entities (protein, cell type, disease, etc.) or more complex relations (e.g. interaction, transport, regulation, etc.). A class mention can have attributes. These attributes are represented as slot mentions (as a frame can have slots). The current structure uses two types of slot mentions. Complex slot mentions are slots that have other class mentions as their fillers, while non-complex slot mentions are filled by simple Strings.

To illustrate the mention structure described here, let us use as an example the *protein transport* frame (shown below) and the example sentence:

`Src relocated the KDEL receptor from the Golgi apparatus to the endoplasmic reticulum.`
(PMID: 12975382)

- protein transport
  - transported entity: The protein being transported
  - transporter: The protein doing the transporting
  - source: The cellular component where the transport event begins
  - destination: The cellular component where the transport event ends

The mention of *protein transport* in the example sentence above can be represented using the following procedure:

1. Create an annotation for the text "Src." Link this CCPTextAnnotation to a CCPClassMention of type *protein*. Add to the CCPClassMention a CCPNonComplexSlotMention of type *Entrez gene ID* with a single slot value of "6714."

2. Create an annotation for "KDEL receptor" with a *protein* class mention containing an *Entrez gene ID* slot filled with "10945"

3. Create an annotation for "Golgi apparatus" with a class mention of type *Golgi Apparatus*.

4. Create an annotation for "endoplasmic reticulum" with a class mention of type *Endoplasmic Reticulum*.

5. Create the *protein transport* annotation. This annotation will have four CCPComplexSlotMentions, one for each slot: *transported entity*, *transporter*, *source*, and *destination*. The fillers for the four complex slot mentions will be the class mentions created earlier.

The generated *protein transport* class mention structure for this example is shown below.

- class mention, name=*protein transport*
  - complex slot mention, name=*transported entity*
    - class mention, name=*protein* “KDEL receptor”
      - non-complex slot mention, name=*Entrez gene ID*, value=“6714”
  - complex slot mention, name=*transporter*
    - class mention, name=*protein* “Src”
      - non-complex slot mention, name=*Entrez gene ID*, value=“10945”
  - complex slot mention, name=*source*
    - class mention, name=*Golgi Apparatus* “Golgi apparatus”
  - complex slot mention, name=*destination*
    - class mention, name=*Endoplasmic Reticulum* “endoplasmic reticulum”

CCPMention [edu.uchsc.ccp.uima.mention]
The superclass for all CCP Mentions (class mention, complex slot mention, and non-complex slot mention).

- *MentionName <String>*: The name of this mention.

CCPClassMention [edu.uchsc.ccp.uima.mention]
The CCPClassMention is the root of a flexible class structure that can store virtually any frame-based representation of a particular class. Common class mention types include, but are not limited to, such things as entities (protein, cell type, cell line, disease, tissue, etc.) and frames (interaction, transport, regulation, etc.).

- *SlotMentions <FSArray:CCPSlotMention>*: A class mention optionally has slot mentions which represent attributes of that class. There are two types of slot mentions, complex and non-complex. The difference between complex and non-complex slot mentions is simply the type of filler (or slot value) for each. Complex slot mentions are filled with a class mention, whereas non-complex slot mentions are filled by simple Strings.

- *CCPTextAnnotations <FSArray:CCPTextAnnotation>*: Just as CCPTextAnnotations are linked to a CCPClassMention, it is sometimes useful to be able to follow a CCPClassMention back to its corresponding CCPTextAnnotation, therefore, this FSArray contains links to the CCPTextAnotation(s) for this class. [NOTE: The use of an array is probably not needed here as there is typically only one occupant in the FSArray. This may be addressed in a future release.]

CCPSlotMention [edu.uchsc.ccp.uima.mention]
The superclass for all slot mentions (complex and non-complex).

CCPComplexSlotMention [edu.uchsc.ccp.uima.mention]
A slot mention is deemed "complex" when its slot filler is a class mention as opposed to a String (See non-complex slot mention for String fillers). An example of a complex slot mention is the *transported entity* slot for the protein-transport class which would be filled with a protein class mention.

- *ClassMentions*: The class mentions which are the slot fillers for this complex slot.

CCPNonComplexSlotMention [edu.uchsc.ccp.uima.mention]
The non-complex slot mention has slot values which are constrained to be Strings. An example of a non-complex slot mention would be the *Entrez_Gene_ID* slot for a gene class mention that might be filled with the String "12345"

- *SlotValues <StringArray>*: Slot fillers (values) for this non-complex slot mention.

Document Annotation Class Descriptions

CCPDocumentAnnotation [edu.uchsc.ccp.uima.annotation]
This is the superclass for all document-level annotations (CCPDocumentInformation, CCPDocumentSection, CCPDocumentSubSection).

CCPDocumentInformation [edu.uchsc.ccp.uima.annotation]
The CCPDocumentInformation annotation includes document metadata such as the document ID, document collection ID, secondary document IDs, document size, etc.

- *DocumentID <String>*: The document ID is a String representing a unique identifier for a particular document within a particular document collection.
- *DocumentCollectionID <Integer>*: The document collection ID is an Integer that uniquely identifies a particular document collection.
- *DocumentSize <Integer>*: The size of a document is logged as the number of characters it contains.
- *SecondaryDocumentIDs <StringArray>*: This StringArray is used for secondary document ID storage. For example, in the biomedical domain, a particular document might be associated with a PubMed ID, however it might also have a deprecated Medline ID, or perhaps a PubMed Central ID, either of which could be stored in this StringArray. It is recommended that the type of ID along with the ID itself be stored, e.g. “MedlineID:12345”.

CCPDocumentSection [edu.uchsc.ccp.uima.annotation]
The Document Section annotation allows for an entire document section to be annotated as such, and stores section-specific information such as the section identifier.

- *SectionID <Integer>*: The section ID for this document section, e.g. 1 = “Title” 2 = “Abstract”
- *SectionLength <Integer>*: The length of this document section (in characters).
- *SectionName <String>*: The name of this document section, e.g. Title, Abstract, Introduction, etc.
- *SubSections <FSArray:CCPDocumentSubSection>*: Allows for direct linking to CCPDocumentSubSections residing in this document section.

CCPDocumentSubSection [edu.uchsc.ccp.uima.annotation]
This annotation type is used to annotate document subsections.

- *SectionName <String>*: The name of the document subsection.

Explicitly-Defined Syntactic Annotation Class Descriptions

CCPTokenAnnotation [edu.uchsc.ccp.uima.annotation.syntactic]
This annotation type is used to mark up tokens.

- *PartOfSpeech* <*String*>: The part of speech symbol is stored here. (In the future, a field denoting the tag set used should probably be added to the CCPTokenAnnotation class.)
- *Lemma* <*String*>: The token lemma.
- *Stem* <*String*>: The token stem.
- *TokenNumber* <*Integer*>: The token number indicates the placement of the token within the document text.
- *TypedDependencies* <*FSArray:CCPTypedDependency*>: If this token is known to be involved in any typed dependency structures, then they are linked through this FSArray.

CCPTypedDependency [edu.uchsc.ccp.uima.annotation.syntactic]
This typed dependency structure was created to hold typed dependency information generated by the Stanford Parser[2,3].

- *GovernorTokenNum* <*Integer*>: From the Stanford Parser documentation: The governor token "describes the governor (regent/head) of the dependency relation."
- *DependentTokenNum* <*Integer*>: From the Stanford Parser documentation: The dependent token "describes the dependent (argument/modifier) of the dependency relation."
- *Relation* <*String*>: From the Stanford Parser documentation: The relation "names the type of dependency (subject, instrument, ...)."

CCPPhraseAnnotation [edu.uchsc.ccp.uima.annotation.syntactic]
This annotation type is used to annotate phrases within the document text.

- *PhraseType* <*String*>: The phrase type symbol (e.g. NP) is stored here.

CCPClauseAnnotation [edu.uchsc.ccp.uima.annotation.syntactic]
This annotation type is used to annotate clauses within the document text.

- *ClauseType* <*String*>: The clause type symbol is stored here.

CCPSentenceAnnotation [edu.uchsc.ccp.uima.annotation.syntactic]
This annotation type is used to annotate sentences within the document text.

References

1. Philip V. Ogren Knowtator: A plug-in for creating training and evaluation data sets for Biomedical Natural Language systems. *In Proceedings of the 9th Intl. Protg Conference*, 2006

2. Dan Klein and Christopher D. Manning. 2002. Fast Exact Inference with a Factored Model for Natural Language Parsing. *In Advances in Neural Information Processing Systems* 15 (NIPS 2002), December 2002.

3. Dan Klein and Christopher D. Manning. Accurate Unlexicalized Parsing. *Proceedings of the 41st Meeting of the Association for Computational Linguistics*, 2003.

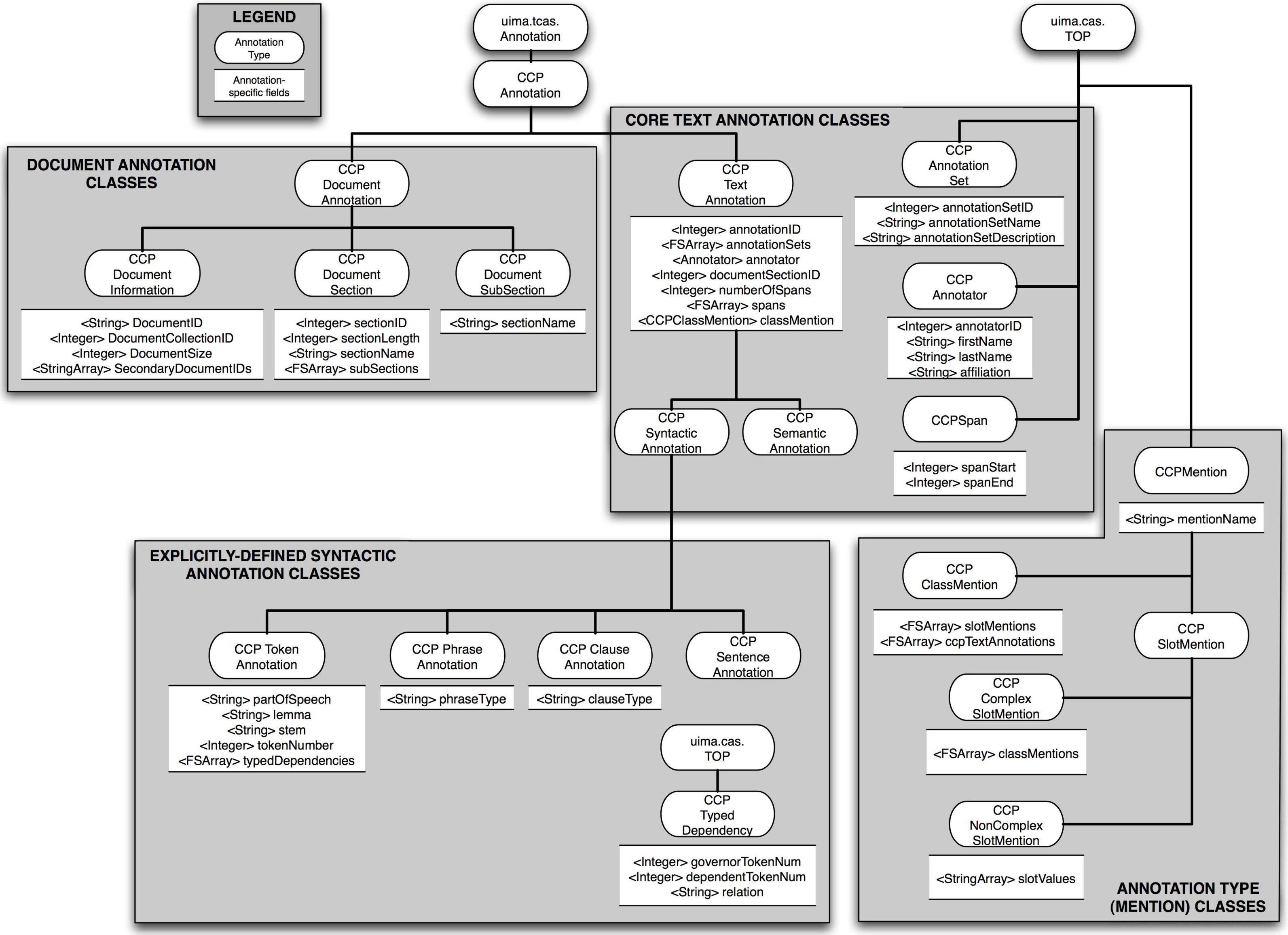

Figure 1: The Center for Computational Pharmacology UIMA Type System
